# Wildlife Population Welfare as Coherence Between Adapted Capacities and Environmental Realities: A Case Study of Threatened Lamprey on Vancouver Island

**DOI:** 10.3389/fvets.2018.00227

**Published:** 2018-09-24

**Authors:** Craig Stephen, Joy Wade

**Affiliations:** ^1^Canadian Wildlife Health Cooperative, Western College of Veterinary Medicine, University of Saskatchewan, Saskatoon, SK, Canada; ^2^Fundy Aqua Services Inc., Nanoose Bay, BC, Canada

**Keywords:** welfare, lamprey, health, conservation, harm reduction, endangered species, resilience

## Abstract

Wildlife conservation lacks a well-accepted ethical foundation for population welfare. In this paper we propose a definition of wildlife population welfare and use a case study to suggest its value for species recovery planning. We define wildlife population welfare as coherence between the species' adapted capacities and the realities of its current environment. We present a case study of the Cowichan Lake lamprey (*Entosphenus macrostomus*), a parasitic fish species endemic to three connected lakes in British Columbia, Canada. Individual-level welfare concerns were insufficient to inspire actions to protect this threatened species. The key threats to Cowichan Lake lamprey can be linked to anthropogenic changes and global threats such as climate change. Due to prevailing uncertainties and the inability to eliminate critical threats, the species recovery plan was focussed on securing critical environmental and social assets to meet evolved adaptations of lamprey while considering the needs of other species, including people. This assets focussed approach was well suited to developing consensus for action to enable a harm reduction perspective that recognizes that many of the threats cannot be eliminated but actions could be taken to enable the population to succeed by protecting critical environmental resources. This was consistent with our population welfare perspective which focusses on assets rather than deficits to help identify shared priorities for species recovery, conservation obligations, and social expectations.

## Introduction

There is little doubt that human activities are harming wild animals^1^ (1). The plethora of reports of species declines and extinctions create innumerable conservation challenges. While we like to think that conservation priorities and actions are objective and science based, human attitudes and values shape our conservation behaviours (2). Which populations to protect and when to intervene is a matter of choice. Kirkwood and Sainsbury (3) identified four factors that influence our attitudes toward wildlife; (i) the extent to which we are responsible for harm to them; (ii) the extent to which the harmed animals are under our stewardship; (iii) the severity of the problems that harm wildlife and (iv) cultural and economic factors, including the popularity of the species involved. The authors noted the illogical but heavily weighted role popularity plays. Sociopolitical considerations, resource limitations, and ethical concerns further dictate which species can be protected and when conservation actions are implemented (4). It is increasingly accepted that conservation should not come at the expense of individual animal welfare, yet a well-accepted and applied ethical foundation for wildlife conservation that considers animal welfare is lacking (5). This is due in part to the different “value lenses” used by animal welfare and conservation scientists, with the former valuing the health, quality of life and affective states of individuals and the latter focused on ensuring the sustainability and integrity of populations and ecosystem diversity (6).

## Background

Animal welfare and conservation have found a common ground in guidelines for the ethical use of wildlife in research and management (7), but there remains a gap when we attempt to find a shared vision for success at a population level. Conservation and animal welfare share the desire to prevent harm to animals (8). To harm something or someone means to damage them or make them less effective or successful than they were. Organizations such as the Canadian Council on Animal Care (9), have developed animal welfare guidelines that are damage focused and intend to reduce harm by minimizing stress to individuals and discouraging procedures that have lasting negative population effects or affect the species' existence. There is less guidance on how to avoid harms that make a wild species “less effective or successful.” In some settings, conservation is deemed successful if measures are no longer necessary to prevent extinction (10). Others suggest that avoiding extinction is far too low of a threshold for success and advocate that conservation should promote self-sustaining, diverse, healthy, and resilient species (11). Ultimately, how we assess population level welfare is context dependent (12) and the current context wildlife is facing is that unprecedented global socio-ecological changes are depriving wildlife from the resources needed to prevent harm and be successful (5, 13).

The 2016 Living Planet Index clearly links the 48 to 66 per cent decline in the more than 3,700 wild species assessed between 1970 and 2002 to anthropogenic factors including habitat degradation, invasive species, climate change, pollution, unsustainable freshwater use, and species overexploitation (14). Economic growth that drives these mega-trends is the limiting factor for wildlife welfare (5). Trade-offs between conservation and human use of ecosystem goods and services require compromise between the needs for conservation, ecosystem functioning and resilience, and human livelihoods (15). Finding a shared perspective that allows for concomitant consideration of wildlife welfare and human well-being is becoming an increasingly important endeavour to facilitate actions to protect wildlife in the face of scientific uncertainty and social conflict.

Conservationists unavoidably find themselves grappling with difficult and conflicting social and economic issues that impede actions to secure critical resources that meet the evolved needs and social expectations for wildlife (16). The salutogenesis concept derived from human well-being literature (17) may help bridge conservation and wildlife welfare to inspire actions on the major threats to wildlife. This approach asks why an individual, group, or community stays well despite stressful situations and hardships. Rather than focusing on obstacles and deficits, it deals with securing critical resources to stay well. It is consistent with the concept of harm reduction which promotes actions to build socio-ecological resilience in individuals and populations in the face of uncertainty and social conflict (13). The salutogensis concept of a “sense of coherence” (which reflects the coherence between the capacity to identify, benefit, and use resources to deal with stress and the reality of current living conditions) is consistent with (18) conceptual model which sees animal welfare compromised when adaptations possessed by the animal make an imperfect fit to the challenges it faces in the circumstances in which it lives.

In this paper, we propose a definition of population welfare as coherence between the adapted needs of a species with critical social and environmental resources. We use a case study to illustrate how this definition is applicable to species recovery planning that can inspire positive attitudes to conservation and the development of recovery plans that address the mega-trends that drive many of the harms to wildlife.

## Discussion

Cowichan Lake lamprey (*Entosphenus macrostomus*) is an extreme endemic freshwater parasitic fish species found only in Cowichan, Bear and Mesachie lakes in British Columbia, Canada. These three lakes are hydrologically connected; the watershed has a catchment area of 930 km^2^, less than half of which is attributed to Cowichan Lake, one of the largest bodies of freshwater on Vancouver Island (6,204 ha area) (19). The outflow of Cowichan Lake is regulated through a weir which has supplied water since 1957 via the Cowichan River to meet the socio-economic and ecological needs of the watershed.

In 2003, Cowichan Lake lamprey was listed as Threatened under Canada's *Species at Risk Act* (SARA). A recovery strategy for the species was completed in 2007 (20). The basic biology of Cowichan Lake lamprey such as longevity, feed preference, spawning, and rearing requirements is largely unknown. This is mostly due to them only being recently discovered, highly cryptic and of no commercial or recreational value. It is recognized that they are an integral part of the ecosystem, like any other species, and have significant scientific value however, these animals are often not well-regarded publicly as they are a parasitic species that feeds on socially highly valued salmonids. The reputation of the Cowichan Lake lamprey has been further tainted by stories of the effects of invasive sea lamprey (*Petromyzon marinus*) on valuable fisheries in the Great Lakes (21) and by popular media depicting lamprey as “aquatic vampires.” Despite their protected status, stories of fishers killing these animals or public distain for this species are common. It is likely that this species will always remain at some risk due to its extremely limited distribution (20, 22). The many unknowns and persistent risk to this uncharismatic species present challenges in promoting actions to protect the welfare of the population.

The most imminent threats to Cowichan Lake lamprey are water use and climate change both individually and cumulatively (20) and destruction of critical habitat (22). In recent drought years, plans have been approved for the emergency draw down of Cowichan Lake below historical levels to supply freshwater for the operation of a wood mill. Emergency draw downs take place in the fall after all other water resources stored in the lake are exhausted. This practice harms lamprey as it reduces available spawning and early rearing habitat. Updated climate models for the region indicate that if no changes are made to water storage and water use is maintained at current levels, these conditions will result in reduced access to spawning grounds and larval rearing habitat, decoupling the evolved needs of this species with its current environment. This has already been documented in a drought year (23). While one might conclude that this species is resilient enough to withstand periodic droughts as they have persisted in this system since the last glaciation (24), the anticipated new “normal” of repeated droughts, coupled with increased water use and decreased riparian habitat due to foreshore development may not be consistent with its adapted capacity. Lack of freshwater in the lake also affects other downstream uses including waste water management, salmon conservation, agriculture irrigation and recreational uses.

In recovery planning for the species it has been recognized that; (i) a target abundance is not currently possible to calculate due to the many unknowns about its biology; (ii) the inherent ecological value of this species is not sufficient to motivate conservation actions among some user groups as it is not recreationally or commercially important; but (iii) the primary threats to Cowichan Lake lamprey are not unique to this species. An additional reality is that protected species such as Cowichan Lake lamprey receive much less funding and effort than other more charismatic species such as BC's southern resident killer whales (*Orcinus orca*).

In the absence of specific biological targets for recovery planning, those working toward this species' recovery by necessity, focused on the environmental and social resources to meet the adapted needs of the species. The population welfare approach was, therefore, reflected in the species recovery plan which has the objectives (20, 22) of: (i) maintaining a self-sustaining population that is resilient to short-term habitat perturbations (ii) maintaining, and where possible enhancing, the ecological integrity of lamprey habitat; (iii) increasing scientific understanding through additional investigation of taxonomic status, natural history, critical habitat and threats to the species' persistence and; (iv) fostering awareness of the species and its conservation status, and encouraging active local involvement in stewardship and habitat protection. The recovery plan further recognizes that activities aimed at protecting and enhancing other species of fish and wildlife are likely to also benefit Vancouver lamprey, and vice versa (20).

Concurrent to recovery attempts for this species is the development of a Cowichan Water Use Plan that aims to accommodate the many ecological, social, and economic needs being threatened by impacts on freshwater habitats. The planning process is a partnership between the local Regional District government, Aboriginal communities, industry, and a multi-stakeholder Watershed Board. It aimed to determine better use of water resources which are sustainable and can meet future demands under climate change conditions. The needs and threats to Cowichan Lamprey have now been taken into consideration in the drafting of the Water Use Plan; most notably, the requirement of water during the summer for spawning and early rearing of eggs and larvae.

Bringing this species into the Water Use Plan has increased community awareness of the requirements of this species as well as highlighted the conservation, recreational, and resource use value of directing recovery actions to critical resources shared by lamprey, people and other species such as benthic invertebrates, amphibians, fish and other aquatics animals co-habiting the lamprey's niche. It is now recognized that activities aimed at protecting other wildlife species will likely benefit Cowichan Lake lamprey (20). Further progress to address data gaps to identify determinants of population welfare including conducting new research to identify critical habitat and completing management activities that help reduce impacts on, and better understand the threats to, Cowichan Lake Lamprey (22). Most recently, the first record of nest building and spawning of Cowichan Lake lamprey was reported (25). This work provides preliminary insights into the habitat and environmental requirements for this critical stage of the lamprey life cycle and has helped inform future research and the Water Use Plan.

Earlier recommended actions for this species included determining traditional fisheries science indices such as species abundance and recovery targets. However, there are significant challenges to estimating the abundance of Cowichan Lake lamprey. For example, it is unknown how spatial variation and capture methods combined with a complicated and undefined life history affect estimates of abundance. In addition, little has been done to determine how to assign thresholds for required numbers and demographics specific to the biological attributes of the species to support self-sustainability. In the face of these unknowns, Cowichan Lake is experiencing more frequent episodes of drought, near-shore users continue to modify the riparian habitat, environmental changes are impacting the abundance of the lamprey's prey, and human population growth places more demands on the ecosystem. The population welfare approach described in this case study promotes actions that would reduce the likelihood that well-documented harms, like climate change and riparian habitat disturbance, would make this species less effective and successful. The collaborative actions associated with this species ecosystem now not only address the population welfare needs of the Cowichan Lake lamprey but also are supporting efforts to identify and address the social resources associated with regional mega-trends. They are also supporting research and monitoring as management activities to minimize harm and achieve the recovery goals.

The recovery strategy acknowledges that protecting this species is a collective responsibility involving multiple levels of government, First Nations and community members. With more frequent applications for draw down permits and growing water use concerns, local community groups have been more active in citizen science and outreach for this species. While the consideration of the lamprey's needs in the water use plan is a critical success, its implementation awaits endorsement by local citizens and governments.

## Concluding remarks

The assets focussed approach to population welfare was consistent with the needs for recovery planning of the Cowichan Lake lamprey. It was better suited to developing consensus for action than a focus on damage to individual animals. It enabled a harm reduction perspective that recognizes that many of the threats to this species cannot be eliminated but actions could be taken to enable the population to succeed by protecting critical environmental resources to meet evolved adaptations while considering the needs of other species, including people. Harm reduction is generally used to describe a set of public health and health promotion strategies to prevent or reduce the adverse consequences to all members of the community rather than only target the hazard. It has been proposed as an approach to promote collaborative policy and action to protect wildlife health by discovering means for horizontal, cooperative actions in advance of serious, irreversible impacts (13). The population welfare perspective presented in this paper provided a bridge between animal welfare, conservation and emerging definitions of wildlife health (26) and provided a foundation for conservation across perspectives and needs. It is consistent with the concepts of one welfare, ecohealth and environmental well-being, all of which serve to foster relationships between people and their ecological system, leading to successful management, distribution, and sustainability of resources for current and future generations as well as for multiple species (27, 28)

In humans there is a close connection between a person's sense of coherence and their health and well-being (29). Key to the salutogeneis concept is that strategies that promote resilience and access to usable critical resources also will contribute to problem reduction and prevention (17). Whereas it is common for estimates of abundance to be a central pre-occupation of fisheries sciences, it may not be suited to conservation science where delays in developing and applying methods to estimate the abundance of understudied or cryptic species will allow ongoing declines of the quality and availability of resources for which they have an adapted dependency for their survival.

Wildlife population welfare as presented in this paper clearly overlaps with core concepts of conservation and population health. In each of these fields, management targets distal determinants of health, welfare, or sustainability by ensuring a species' supporting environment matches its evolved needs. Regardless of the definitions or domains of inquiry used, the perspective used in the case of Cowichan Lake lamprey enabled a; (i) shift away from focussing on estimating a target number in recovery planning; (ii) shifts in attitudes toward action for an uncharismatic species and (iii) support for actions targeting shared critical resources for animal welfare and social well-being. By linking the needs of the lamprey into larger ecosystem management plans, attitudes for species recovery improved and actions were motivated. This is consistent with findings elsewhere that recovery plans for species with greater public or agency profiles are implemented at a higher rate (30).

Successful conservation plans must be clearly linked to species biology as well as attend to the human dimensions of conservation to ensure that recovery plans are appropriately suited to each species' ecological and social situation (31, 32). We propose that population health and welfare may serve as a shared perspective that supports collaborative actions that benefits people while facilitating actions to protect wildlife in the face of scientific uncertainty and social conflict and, therefore, may more likely provoke action, especially for species where charisma and individual animal welfare are insufficient to inspire action.

## Author contributions

Each author contributed equally to this paper. The case study is based on field studies undertaken by JW, the second author and previous conceptual frameworks developed by the first author, CS.

### Conflict of interest statement

JW was employed by the company Fundy Aqua Services which holds no competing interests with this paper. CS declares that the research was conducted in the absence of any commercial or financial relationships that could be construed as a potential conflict of interest.

## Footnotes

^1^For the purposes of this paper wildlife refers to free ranging wild vertebrates, including amphibians, reptiles, birds, mammals, and fish
